# Vertical Handover Prediction Based on Hidden Markov Model in Heterogeneous VLC-WiFi System

**DOI:** 10.3390/s22072473

**Published:** 2022-03-23

**Authors:** Oluwaseyi Paul Babalola, Vipin Balyan

**Affiliations:** Department of Electrical, Electronics and Computer Science Engineering, Faculty of Engineering and the Built Environment, Cape Peninsula University of Technology, Bellville 7537, South Africa; balyanv@cput.ac.za

**Keywords:** hidden Markov model, principal component analysis, radio frequency, received signal strength, vertical handover, visible light communication, WiFi

## Abstract

Visible light communication (VLC) channel quality depends on line-of-sight (LoS) transmission, which cannot guarantee continuous transmission due to interruptions caused by blockage and user mobility. Thus, integrating VLC with radio frequency (RF) such asWireless Fidelity (WiFi), provides good quality of experience (QoE) to users. A vertical handover (VHO) scheme that optimizes both the cost of switching and dwelling time of the hybrid VLC–WiFi system is required since blockage on VLC LoS usually occurs for a short period. Hence, an automated VHO algorithm for the VLC–WiFi system based on the hidden Markov model (HMM) is developed in this article. The proposed VHO prediction scheme utilizes the channel characterization of the networks, specifically, the measured received signal strength (RSS) values at different locations. Effective RSS are extracted from the huge datasets using principal component analysis (PCA), which is adopted with HMM, and thus reducing the computational complexity of the model. In comparison with state-of-the-art VHO handover prediction methods, the proposed HMM-based VHO scheme accurately obtains the most likely next assigned access point (AP) by selecting an appropriate time window. The results show a high VHO prediction accuracy and reduced mixed absolute percentage error performance. In addition, the results indicate that the proposed algorithm improves the dwell time on a network and reduces the number of handover events as compared to the threshold-based, fuzzy-controller, and neural network VHO prediction schemes. Thus, it reduces the ping-pong effects associated with the VHO in the heterogeneous VLC–WiFi network.

## 1. Introduction

Devices are utilizing more wireless networks due to the rising deployment of the Internet of Things (IoT). For the IoT to maintain its full connectivity potential, a communication infrastructure that will serve as a backbone network is required. Visible light communication (VLC) offers an attractive and economical communication platform for IoT systems and has rapidly evolved as a potential alternative to the congested traditional radio frequency (RF) since it uses low-cost light-emitting diodes and photodiodes for both illumination and communication [1]. Hence, the VLC system saves the extra power that is required in the traditional RF communication system. The VLC system occupies a spectrum between 380 nm to 750 nm, corresponding to a frequency spectrum of 430 THz to 790 THz [2]. The VLC system is encouraging for several applications and has been applied in different environments such as indoor [3,4], outdoor [5], underwater [6], and underground [7] scenarios since it is capable of providing high data rates with limited security issues compared to the RF system with lower bandwidth and security challenges.

For the indoor VLC system, a directed LoS link is the simplest configuration, where the channel of transmission is defined by a highly directed angle of beaming of the transmitter and a narrow FoV of the receiver. The directed LoS links do not interfere with noise from ambient light sources since a narrow FoV is usually used as the transmitter and are not prone to multipath propagation effects, therefore exhibiting a high transmission rate. However, the  LoS links offer limited channel coverage area, making it difficult to provide extended coverage and mobility in the indoor environment. This is due to the need for the alignment between the transmitter and receiver. More so, the links experience blockages caused by obstructions from people’s movement and presence of nearby obstacles. As a result, there is a shadowing effect, which reduces the received signal strength (RSS) in the shadowed region and lowers the quality of communication. Moreover, blockage and shadowing issues in the indoor VLC system can be alleviated using a non-directed LoS link or a dispersed topology [8]. Unlike directed LoS links, these configurations employ wide beam transmitters, wide FoV receivers, and surface dispersions to expand the coverage region and achieve mobility in the indoor environment. The non-directed LoS link depends on several reflections from object surfaces, allowing a PD to detect considerable amount of the transmitted light from various directions. On the other hand, diffused design does not involve establishing LoS link (non-directed NLoS) or aligning the wide beam transmitter with the receiver since the emitted light beams are reflected from several surfaces. Nevertheless, non-directed LoS and diffused configuration utilize greater transmit power and are more susceptible to multipath propagation effects such as multipaths-induced dispersions, occurring in the form of ISI. Consequently, degrading the achievable data rate of the links.

Some articles, such as [9,10], have focused on environmental modeling and theoretical analysis of the shadowing effect using different scenarios. Moreover, integrating the VLC system with the RF system helps to avoid service disruption and ensure continuous transmission while maintaining a high data rate in the VLC system [10]. However, a major issue in a hybrid VLC–RF system is the decision to perform either a horizontal handover (in a homogeneous system) or vertical handover (VHO) (in a heterogeneous system). This is because a user device is able to switch from an AP with reduced coverage and/or degraded channel quality to another with stronger connection. For VHO decisions, a PVHO algorithm based on two fundamental VHO schemes, IVHO and DVHO for hybrid VLC and LTE systems, was presented in [11]. In the IVHO scheme, MTs switch immediately during channel disruption while the DVHO scheme sets fixed dwelling time as the duration of short interruption to avoid ping-pong effects. As an improvement to the static DVHO scheme, [12] introduced a CAD-VHO scheme for the VLC-RF system. Note that excessive switching between the APs may result in increased signaling overhead and latency.

As discussed in [13], MT activities are key contributors to VLC disruption, which often result from user mobility and follow predictable patterns. These patterns can be modeled by parameters such as RSS, location, time of day, and duration. In addition, the duration of the VLC–LoS link blockage is usually for a short period of time, requiring minimal handover delay time before service is restored. Hence, it is possible to model and make VLC link interruption prediction using the MT activity record. The use of machine learning in the handover decision process was investigated in [14,15,16,17]. In [14], a dynamic coefficient training through ANN was adopted to provide reliable communication for group mobility and improve the handover decision process. More so, an LVQNNs technique for maintaining steady communication based on the RSS indicator, data rate criterion, service cost, and statistics of the MT’s speed was suggested in [15]. The authors in [16] presented a context-aware VHO in a heterogeneous WiMax and WiFi system. The Whale optimization algorithm was implemented with the neural network to ensure effective handover prediction using the RSS indicators. In [17], a VHO algorithm was presented for heterogeneous wireless networks based on multi-attributes and NN. The download rate of the integrated network was utilized as a prediction target, while the appropriate wireless network for VHO decision was selected by the NN algorithm. This method presented a tradeoff between the handover success rate and the number of wireless networks in the integrated system. In addition, fuzzy prediction is a well-known intelligent method for enhancing VHO decisions in the heterogeneous networks. This approach depends on mobility prediction to compute the declination ratio of expected signal output to the actual signal output. A fuzzy controlled VHO system was developed for heterogeneous networks in [18]. The authors utilized a multi-criteria approach to address network selection issues. In addition, the fuzzy-logic-based technique was utilized to obtain seamless VHO in [19]. The handover technique reduced ping-pong effects while improving the overall performance for mobile users in the network. Similarly, a vertical handover decision algorithm was proposed for next-generation networks using a fuzzy predictive model in [20]. The scheme utilized a multi-layer feed forward network to determine the user’s next position and a multiple-attribute access network selection function to obtain the most likely access network.

Furthermore, hidden Markov model (HMM)-based HHP schemes for user mobility in wireless networks were presented in [21,22,23]. These schemes were developed to provide horizontal handover between two or more APs in the same network. The authors in [21] employed the HMM to predict RSS values from observed users’ movement data. A fuzzy controller module was further utilized to classify the handover decision before deciding whether or not to execute the handover. In addition, an approach to improve the handover decision in femtocell networks based on HMM was suggested in [22]. The HMM predicted the next AP position based on the geographical locations of the previous and current users. Similarly, [23] utilized the HMM scheme to predict service eNodeB in the long-term evolution (LTE) network according to user mobility for improved communication. The handover approach relied on trajectory features to trigger handovers between two non-adjacent APs. The model exploited a large number of hidden states, leading to more inaccurate sequences and increased computational complexity.

Moreover, other studies have considered formulating heterogeneous handover prediction as MDP problems. In [24], a VHO scheme for heterogeneous VLC and WiFi networks was formulated as an MDP problem. The technique adopted a dynamic approach to achieve a trade-off between the cost of switching and the need for delay. The scheme made VHO decisions according to the length of the queue and the state of the wireless channel. A VHO scheme to enhance the user’s QoE in an indoor VLC environment was investigated in [25]. Likewise, the scheme was formulated as an MDP problem that maximizes the QoE of users and reduces handover delay. The decision to perform VHO was based on the current location of the user and the mode of transmission, which could either be a VLC or an RF channel. In [26], a VHO technique for an indoor hybrid VLC–Femto system was considered based on candidate decisions. The technique utilized AHP and CG to manage an MADM framework and for matching different types of traffic. The rationality of each decision was computed by combining the results from AHP and CG to select the one with a higher degree. A larger number of hidden states was exploited by the models and thus leading to extra inaccurate sequences and increased computational complexity.

In this article, an automated vertical handover prediction algorithm for a heterogeneous VLC–WiFi system is developed to improve communication in the indoor environment. The HMM is employed as a tool to model the disruption (OFF) and continuation (ON) of the VLC–LoS link. The proposed VHO utilizes the RSS dataset of the VLC and WiFi, containing RSS values that correspond to the MTs’ location. RSS values are time-varying due to the blockage in the VLC–LoS download link and the instability of the WiFi system. Unlike other existing HMM methods, effective RSS values are obtained from the huge RSS datasets using a PCA method, thus reducing the computational complexity of the HMM. The extracted features are then restructured into window sizes at different locations, which are employed with the HMM to maximize the handover decision-making process. The results of this paper are unique since the proposed VHO reduces excessive switching in a short period of time known as the ping-pong effect and yields an enhanced dwell time compared to existing state-of-the-art VHO schemes.

The remainder of this article is arranged in the following manner. Section 2 discusses the system model, method of data collection and feature extraction and basic HMM principles. Section 3 demonstrates the design of the dual HMMs for predicting the next RSS value and optimizing handover decisions. Section 4 presents performance evaluation of the proposed VHO scheme based on HMM. The Section evaluates the HMM prediction accuracy, the number of handovers in the system, and the dwell time. Section 5 concludes the article.

## 2. Methodology

### 2.1. VLC Network Model

In this article, a direct VLC–LoS propagation link is modeled due to its power efficiency dominance over the NLoS reflected links. The VLC channel quality relies on LoS transmission and cannot guarantee continuous transmission when there is a blockage and/or the MT is not in the VLC range. We consider the VLC system in an indoor environment with LED bulbs (transmitter), having a mixture of 475 THz, 535 THz, and 638 THz. Each LED, having 30 V peak voltage and 300 mA driving current is placed at the ceiling center in a 3 m × 3 m space. The VLC receiver is a low-cost (S5971) Silicon PIN PD, which offers good temperature tolerance, fast response, and wideband characteristics at a low bias. The LED is situated at 1.5 m above the upward facing PD attached to a user’s device.

Suppose a flat frequency response around the DC transmitter and receiver. The DC gain of the LoS channel is computed as [8]:

(1) $$H=\left\{\begin{array}{cc} \frac{(m+1)A}{2\pi d_{r,t}^{2}}\cos^{m}\left(\vartheta_{r,t}\right)\cos\left(\theta_{r,t}\right)g\left(\theta_{r,t}\right)c\left(\theta_{r,t}\right), & 0\leq \theta_{r,t}\leq \vartheta_{c}\\ 0, & \theta_{r,t}>\vartheta_{c}, \end{array}\right.$$

where $d_{rt}$ is the Euclidean distance between the AP and *r*-th user, $R=\frac{\left(m+1\right)}{2\pi }\cos^{m}\left(\vartheta_{r,t}\right)$ represents the Lambertian radiant intensity, $\vartheta_{r,t}$ is the radiation angle related to the axis normal to the VLC AP plane, and $\theta_{r,t}$ represents the incidence angle with reference to the axis normal to the PD. Note that the PD’s FoV = $\vartheta_{c}$ is determined by the maximum incident angle. More so, *A* is the PD’s physical area, $m=-\ln\left(2\right)/\ln(\cos\left(\vartheta_{1/2}\right))$ denotes the order of Lambertian emission, while $g(\theta_{r,t})$ and $c(\theta_{r,t})$ are the optical filter and concentrator gains, respectively. Consequently, the relationship between the LED-transmitted power ($P_{l}$) and optical power of the receiver ($P_{pd}$) is obtained as [8]:

(2) $$P_{pd}=HP_{l}.$$

In addition to the indoor LoS channel model, optical channel noise is considered in the network model. Optical background noise sources in the indoor VLC systems include ambient light noise, signal and ambient light induced noise in the PD, and thermal noise produced by the TIA [8]. In this study, the VLC channel is modeled as a linear optical AWGN channel. The PD current *I* is given as [27]:

(3) $$\begin{array}{c} I=\eta P_{l}⊗H+N, \end{array}$$

where η denotes the photo sensitivity of the PD, ⊗ symbol represents convolution, and *N* is the noise, which is the combination of the shot noise and thermal noise, that is:

(4) $$\begin{array}{c} N=N_{shot}^{2}+N_{thermal}^{2}. \end{array}$$

The signal and ambient light induced shot noise variance is given by [13]:

(5) $$\begin{array}{c} N_{shot}^{2}=2qR\left(p_{r}+p_{n}\right)B, \end{array}$$

where *q* is the electronic charge, *R* represents the PD responsivity, $p_{n}$ denotes the received noise power, $p_{r}$ defines the received optical power, and *B* indicates the receiver bandwidth. On the other hand, the thermal noise variance is expressed as [13]:   

(6) $$\begin{array}{c} N_{thermal}^{2}=\frac{4KT}{BR_{f}}, \end{array}$$

where *K* denotes the Boltzmann’s constant, *T* represnts the absolute temperature, and $R_{f}$ defines the TIA feedback resistance.

Table 1 shows the VLC system parameters used in this study. Similar to [13], a value of $60^{\circ }$ is allocated to both the direction of the LED, defined by a semiangle of the power, $\vartheta_{1/2}$, and the direction of the PD, specified by its FoV. In addition, constant values are assigned to $g(\theta_{r,t})$ and $c(\theta_{r,t})$ to avoid their dependence on the incidence angle. A fluorescent lamp that operates at 50 Hz is used to represent the artificial ambient light noise, while a visible passband filter with 660 nm wavelength is placed in front of the PD to prevent other wavelenghts. The PD functions at a wavelength of 660 nm and converts measured light to electrical current at a cutoff frequency of 100 MHz. Furthermore, the low noise TIA (AD8015) amplifies the input current from the PD to an output voltage signal. Other undesired noise components of the transmitted optical signal are filtered using an electrical low pass Bessel filter. The demodulator is used to recover the original transmitted data.

### 2.2. VLC–WiFi Network Model

**Figure 1:**
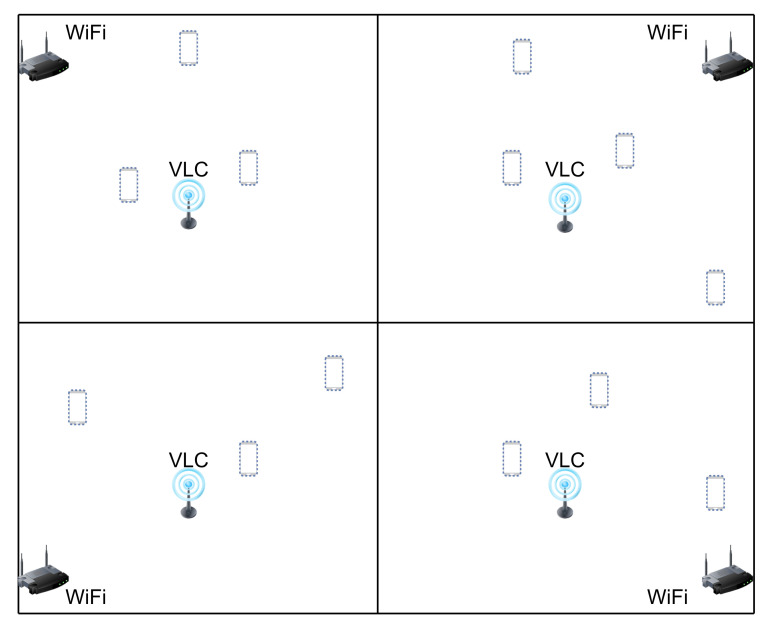
Hybrid VLC-WiFi Network Model.

We consider an indoor environment consisting of a hybrid VLC–WiFi system. Figure 1 shows a typical indoor scenario where one VLC AP is placed on the ceiling in each room and a single WiFi AP (always on) is deployed in a corner of the rooms. The VLC has low coverage of about 2–3 m but provides high bandwidth. However, the WiFi provides lower bandwidth and covers a wider area (overlapping the VLC AP) in the apartment. Suppose several users holding multi-mode MTs randomly move around in the apartment. A vertical handover is expected to be prompted whenever there is a blockage or disruption in the VLC link of the MTs.

This study focuses on the downlink transmission in the heterogeneous VLC–WiFi network. This implies that VLC is used as the preferred downlink since it has higher bandwidth. Moreover, the WiFi network is designated for uplink transmission and can also serve as a backup if the VLC–LoS downlink channel is blocked or when the VLC is out of range. This is because the WiFi provides a wider coverage and can penetrate opaque objects.

In a real-world scenario, the location of both the VLC and WiFi APs can be irregular. However, for the purpose of simplicity and to avoid interference among the VLC APs, we suggest the arrangement shown in Figure 1. A CSMA/CA [28] is used to prevent interference between WiFi APs. More so, it is essential to allocate resources efficiently in a real-time VLC–WiFi system, especially when the receiving system is saturated. Moreover, it is assumed in this study that data can be delayed if there is an interruption or/and shadowing during VLC–LoS downlink transmission. The delayed data will be re-sent when the VLC channel becomes available. Otherwise, communication can proceed over the available WiFi network. As a result, an automated VHO prediction scheme is introduced to effectively manage the decision-making process in the hybrid system. This is accomplished by minimizing both switching costs and optimizing the dwell time of the VLC–WiFi system.

### 2.3. Mobility and Blockage Model

Similar to [21], RSS values were measured by an MT moving in the indoor apartment at a speed between 1 m/s and 1.5 m/s. The measurements were collected at a sampling rate of 100 Hz for a duration of 500 s from 6 history locations over short distances. The user mobility was simulated using the random waypoint model [29], which ensures that users travel in a straight line from one waypoint to another and that the waypoints are chosen at random in the space. Note that the measured RSS values are directly related to the distances between the MT and the APs. This implies that the closer an MT is to the AP, the stronger the RSS values and vice versa. Strong RSS values were produced when the MT was within 1 m of the VLC AP and there was no blockage on the LoS link. On the other hand, the weak RSS values were obtained when the distance between the MT and the VLC AP was greater than 2 m and the VLC–LoS connection was blocked. This implies that the RSS values are time-varying, and the dimension of the datasets determines the computational complexity of the HMM.

VLC–LoS blockages, such as human crossing, can be modeled using the occurrence and occupation rates over time [10]. The occurrence rate signifies the total number of blockages that occur over a sampling interval, while the occupation rate is the number of times that LoS blockages occur in the network. The blockage typically results in VLC signal attenuation, leading to a significant decrease (negative peak) in the value of the RSS readings. In this study, the LoS blockage event is modeled using the rate of occupation since the event is assumed to follow the Poisson point process and be evenly distributed between 0 and 1.

Consequently, effective RSS values were extracted from the RSS datasets using the PCA method. The reduced feature set may be readily observed and processed using various machine learning methods such HMM while preserving as much data as practicable. Other discriminant analysis methods that have been adopted with HMM include LDA or FDA [30] and DMD [31]. Moreover, the PCA is a multivariate method with low computing complexity for reducing the dimensionality of a huge dataset by transforming variables to smaller components while keeping the information in the larger dataset [32]. The mathematical process for the PCA entails the following steps:

(1) **Normalization**. This ensures that each sampling point in a continuous signal contributes evenly to feature analysis. Generally, a significant discrepancy between the large and small RSS values in observation variables of the dataset produces bias at the output. Therefore, normalizing the variables of the dataset mitigates such bias. Supposing $Z_{l}=\left(\begin{array}{cccc} z_{1} & z_{2} & \cdots & z_{i} \end{array}\right)$ is a RSS vector containing *i* sampling points at a specific location *l*, the normalized signal is given as:
  (7) $$\hat{Z_{l}}=\left(\begin{array}{cccc} \frac{z_{1}-\bar{Z_{l}}}{D(Z_{l})} & \frac{z_{2}-\bar{Z_{l}}}{D(Z_{l})} & \cdots & \frac{z_{i}-\bar{Z}}{D(Z_{l})} \end{array}\right)=\left(\begin{array}{cccc} {\hat{z}}_{1} & {\hat{z}}_{2} & \cdots & {\hat{z}}_{i} \end{array}\right),$$
  where $\bar{Z_{l}}$ and D are the mean and standard deviation of $Z_{l}$, respectively.
(2) **Covariance Matrix Computation**. Note that the observation variables in the RSS dataset have a high correlation since the dataset contains repeated values at some MT locations. Thus, the covariance matrix, M, is computed to identify variations between the input data set and the mean. The M is a d×d-dimensional symmetric matrix that contains the covariances for all possible pairs of observation variables. Suppose that the RSS dataset, *C*, has variables $\{l_{1},l_{2},\ldots ,l_{d}\}\in l$, the covariance matrix of *C* is given by:
  (8) $$M\left(C\right)=\left(\begin{array}{cccc} M(l_{1},l_{1}) & M(l_{1},l_{2}) & \cdots & M(l_{1},l_{d})\\ M(l_{2},l_{1}) & M(l_{2},l_{2}) & \cdots & M(l_{2},l_{d})\\ \vdots & \vdots & \ddots & \vdots \\ M(l_{d},l_{1}) & M(l_{d},l_{2}) & \cdots & M(l_{d},l_{d}) \end{array}\right).$$
  The diagonal elements represent the variances of each observation vector, and the elements of M(C) are symmetric with respect to the main diagonal.
(3) **Eigenvectors and Eigenvalues Computation**. The PCs of the RSS dataset are produced using eigenvectors V and eigenvalues F, which are determined from the covariance matrix. SVD is a well known linear algebraic approach for obtaining V and F. The SVD method decomposes M(C) in Equation (8) to yield unitary matrices, L and Z, as well as a diagonal matrix Y with the same dimension as M(C) and non-negative diagonal components in decreasing order, such that:
  (9) $$M\left(C\right)=L^{*}Y^{*}Z^{†}.$$
  Note that entries in the diagonal of Y correspond to F while the columns of Z represent V, which are sorted in order of the F (from highest to lowest). Thus, the PCs are obtained in order of importance as V.
(4) **Feature Vector Construction**. Supposing that *k*, k<d components have the largest variance, that is, the most information based on the PCA, the *k*-dimensional matrix is expressed as:   
  (10) $$Z_{PC}=\left(\begin{array}{cccc} z_{1,1} & z_{1,2} & \cdots & z_{1,k}\\ z_{2,1} & z_{2,2} & \cdots & z_{2,k}\\ \vdots & \vdots & \ddots & \vdots \\ z_{k,1} & z_{k,2} & \cdots & z_{k,k} \end{array}\right).$$
  Hence, the feature vector is obtained as:
  (11) $$\kappa_{l}=\left[{\left(\frac{1}{k}\sum_{l=1}^{k}\left|z_{l}\right|\right)}_{1},{\left(\frac{1}{k}\sum_{l=1}^{k}\left|z_{l}\right|\right)}_{2},\cdots ,{\left(\frac{1}{k}\sum_{l=1}^{k}\left|z_{l}\right|\right)}_{k}\right]=\left[\nu_{1},\nu_{2},\cdots ,\nu_{k}\right].$$

The extracted features, known as effective RSS, $Z_{PCA}$ (containing the most information) are reshaped to smaller time window (*w*) at various locations for the purpose of selecting the required observation frame for HMM, given by:

(12) $$Z_{PCA}=\left(\begin{array}{cccc} \nu_{1,1} & \nu_{1,2} & \cdots & \nu_{1,k}\\ \nu_{2,1} & \nu_{2,2} & \cdots & \nu_{2,k}\\ \vdots & \vdots & \ddots & \vdots \\ \nu_{w,1} & \nu_{w,2} & \cdots & \nu_{w,k} \end{array}\right).$$

The choice of window size is critical to the performance of HMM since it helps to maintain the probability distribution of the transition matrix in the model. Suppose a very small time window is selected, the observation frame becomes too small, which may not be sufficient for the model to make a fair prediction about the RSS variation’s pattern. On the other hand, selecting a very large time window will result in a large observation frame that may contain several user mobility changes in the indoor environment. Therefore, we test different window sizes to select the one that maximizes the MT’s movement in a considerable observation frame using simulation.

### 2.4. Hidden Markov Model

The HMM is a class of PGM with state (hidden) variables, which are estimated through a sequence of output (observable) variables. The HMM is a flexible tool that can easily predict continuous RSS values and classify the handover decision of MTs in the indoor environment. For a sequence $S=\{s_{1},s_{2},s_{3},\ldots ,s_{N}\}$ of *N* hidden states, each state $s_{i}$ of the model is represented as the locations $L_{i}$ of MT in the building at a particular time *t*. More so, the MT’s movement history at a state is depicted by a sequence of *M* observations (emissions) $O=\{o_{1},o_{2},o_{3},\ldots ,o_{M}\}$. This signifies the possible RSS values received by the MT.

Furthermore, for a given sequence of observable variables, the HMM estimates three important parameters Γ=(π,Ω,Φ), where π is the initial start probability, Ω is a N×N transition probability matrix representing the probabilities of switching from state *i* to *j* at a given time, and Φ is is a N×M emission distribution matrix, referring to the probability of obtaining observation symbols $o_{i}$ from a specific state *j*. To maximize the occurrence of RSS values, Ω is obtained by estimating the ML of the HMM parameters from the sequence of the received RSS values using the Baum–Welch algorithm.

## 3. RSS Prediction Based on HMM

**Figure 2:**
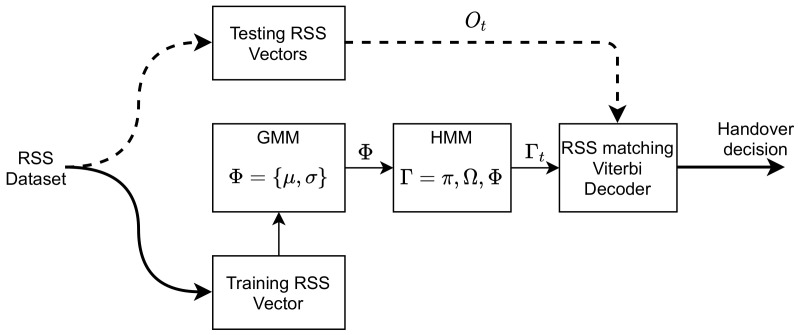
Enhanced VHO prediction system based on HMM.

Figure 2 depicts a typical HMM as an automated scheme for classifying and predicting the next RSS values of the hybrid VLC–WiFi system. This is in contrast to the method in [21], which uses the HMM with fuzzy controller module as a model for prediction.

**Figure 3:**
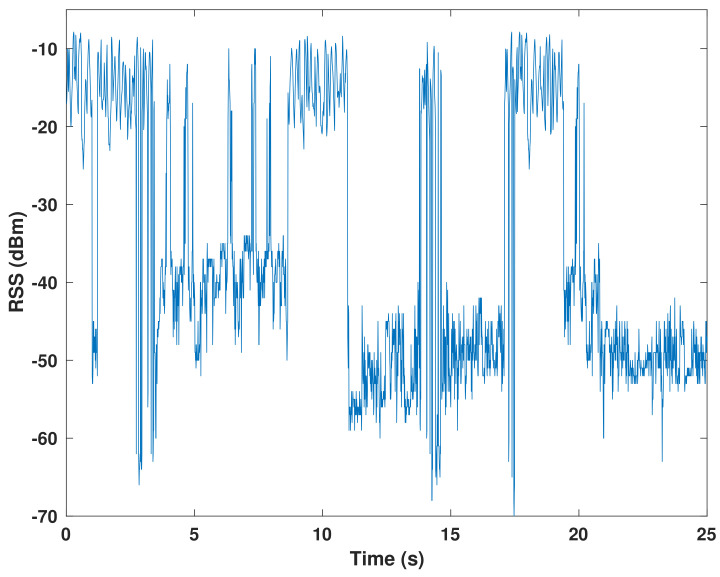
Hybrid VLC–WiFi RSS time series for training.

The RSS dataset is divided into two parts: 70% and 30%, which are used for model training and testing, respectively. Figure 3 shows the time series of the hybrid VLC–WiFi RSS dataset used for training. As mentioned earlier, the VLC signal attenuation is caused by blockage and user mobility. The dataset samples are manually annotated and categorized into three RSS intervals: high, medium, and low intervals as depicted in Table 2. Subsequently, the labeled dataset is used to train the HMM.

Two Gaussian HMMs: “VLC-AP” ($M_{V}$) and “WiFi-AP” ($M_{W}$) models are formed to represent the VLC and WiFi APs, respectively. The initial parameters of each model are given as $M_{V}=\left\{\pi_{v},\Omega_{v},\Phi_{v}\right\}$ and $M_{W}=\left\{\pi_{w},\Omega_{w},\Phi_{w}\right\}$. Here, the sequence of observation *O* for each model is developed to include frames of 3 time slots, which restricts the monitoring of user motion to between 3 and 5 m. Thus, *O* for each category of an RSS indicator is used to train the appropriate HMM.

### 3.1. HMM Training

Training an HMM entails deriving the best parameters of $M_{V}$ and $M_{W}$ to optimize the probability of occurrence of *O* and the joint probability of *O* and *S*, given by:

(13) $$\begin{array}{cc} P(\Gamma ,\Gamma^{old})\; & =\sum_{s}p\left(S|O\Gamma^{old}\right)\ln p\left(OS|\Gamma \right)\\ \; & =\sum_{s_{1}}p\left(s_{1}|O\Gamma^{old}\right)\ln p\left(s_{1}\right)+\sum_{i=2}^{N}\sum_{s_{i-1}s_{i}}p\left(s_{i-1}s_{i}|O\Gamma^{old}\right)\ln p\left(s_{i}|s_{i-1}\right)\\ \; & +\sum_{i=1}^{N}\sum_{s_{i}}p\left(s_{i}|O\Gamma^{old}\right)\ln p\left(o_{i}|s_{i}\right)\\ \; & =\underset{1}{\underset{︸}{\sum_{j=1}^{J}\gamma \left(s_{ij}\right)\ln\pi_{j}}}+\underset{2}{\underset{︸}{\sum_{i=2}^{N}\sum_{n=1}^{J}\sum_{j=1}^{J}\xi \left(s_{i-1,n}s_{i,j}\right)\ln\Omega_{nj}}}\\ \; & +\underset{3}{\underset{︸}{\sum_{i=1}^{N}\sum_{j=1}^{J}\gamma \left(s_{ij}\right)\ln p\left(o_{i}|s_{ij}\Phi \right)}}, \end{array}$$

where $\Gamma^{old}$ is the current estimated parameters, Γ is the parameters to be optimized, $p(S|O,\Gamma^{old})$ is the conditional probability distribution of *O*, $\gamma \left(s_{ij}\right)=p\left(s_{i}|O,\Gamma^{old}\right)$ represents the probability of an MT being in state i=j, and $\xi \left(s_{i-1,n}s_{i,j}\right)=p\left(s_{i-1}s_{i}|O,\Gamma^{old}\right)$ depicts the transitional probability of an MT moving from state *i* to *j*. To solve the optimization problem, an EM algorithm [33] is applied, which iteratively estimates the ML of independent model parameters in the following steps:

**Step 1—Expectation (E):** To evaluate $p(S|O,\Gamma^{old})$, it suffices to compute $\gamma (s_{ij})$ and $\xi (s_{i-1,n}s_{i,j})$ using HMM dependency since several terms vanish from the form of $P(\Gamma ,\Gamma^{old})$ in Equation (13). Therefore, the posterior state probability value $\gamma (s_{i})$ is obtained from a given sequence of RSS indicators using independence of PGM, which is further decomposed to forward $\hat{\alpha }\left(s_{ij}\right)$ and backward $\hat{\beta }\left(s_{ij}\right)$ terms such as:

(14) $$\gamma \left(s_{i}\right)=\hat{\alpha }\left(s_{i}\right)\hat{\beta }\left(s_{i}\right).$$

Both terms can be efficiently computed using the forward and backward algorithm. Furthermore, the transitional probability of an MT moving from a state to another is decomposed using the Bayes’ rule as:

(15) $$\xi \left(s_{i-1}s_{i}\right)=\hat{\alpha }\left(s_{i-1}\right)p\left(o_{i}|s_{i}\right)p\left(s_{i}|s_{i-1}\right)\hat{\beta }\left(s_{i}\right).$$

**Step 2—Maximization (M):** Here, $P(\Gamma ,\Gamma^{old})$ is optimized with respect to Γ={π,Ω,Φ}. Moreover, each parameter in the categories 1, 2, and 3 in Equation (13) is independent and thus can be optimized separately. To maximize π and Ω, we introduce Lagrange functions $f_{1}\left(\pi ,\Upsilon \right)$ and $f_{2}=\left(\Omega ,\Upsilon_{1},\ldots ,\Upsilon_{J}\right)$ considering the constraints that $\sum_{n=1}^{J}\pi_{n}=1$ and $\sum_{r=1}^{J}\Omega_{nr}=1$, r=1,…J. Suppose the derivative of $f_{1}\left(\pi ,\Upsilon \right)$ with respect to $\pi_{j}$ is equal to 0, we have:

(16) $$\pi_{j}=\frac{\gamma_{1j}}{\sum_{n=1}^{J}\gamma_{1n}},\;j=1,\ldots ,J.$$

In addition, let the derivative of $f_{2}=\left(\Omega ,\Upsilon_{1},\ldots ,\Upsilon_{J}\right)$ with respect to $\Omega_{nj}$ be equal to 0:

(17) $$\Omega_{nj}=\frac{\sum_{i=2}^{N}\xi \left(s_{i-1,n}s_{i,j}\right)}{\sum_{r=1}^{J}\sum_{i=2}^{N}\xi \left(s_{i-1,n}s_{i,r}\right)},\;n,j=1,\ldots ,J.$$

Note that the RSS dataset is continuous and follows a normal distribution. Hence, the emission distribution Φ for each AP is a Gaussian distribution, and the probability density function (pdf) is given by:

(18) $$p\left(o_{i}|s_{ij},\Phi \right)=\Lambda \left(o_{i}|\mu_{j}\sigma_{j}\right),$$

where $\Phi =\{\mu_{j},\sigma_{j}\}$, j=1,…J. In addition, Φ is maximized by defining the Lagrange function $f_{3}\left(\Phi ,\Phi^{old}\right)$ such that its derivative with respect to $\Phi =\{\mu_{j},\sigma_{j}\}$ is equal to zero. Hence, the estimated parameters are obtained as:

(19) $$\mu_{j}=\frac{\sum_{i=1}^{N}\gamma_{ij}o_{i}}{\sum_{i=1}^{N}\gamma_{ij}},\;j=1,\ldots J,$$

and

(20) $$\sigma_{j}=\frac{\sum_{i=1}^{N}\gamma_{ij}\left(o_{i}-\mu_{j}\right){\left(o_{i}-\mu_{j}\right)}^{T}}{\sum_{i=1}^{N}\gamma_{ij}},\;j=1,\ldots J.$$

Subsequently, the trained $M_{V}$ and $M_{W}$ models are concatenated to a single model $\Gamma^{\prime }$ using their respective estimated parameters. The trained initial state probability is given as $\pi^{\prime }=\left[\pi_{v},\pi_{w}\right]$, the trained transition probability matrix $\Omega^{\prime }$ is specified by a block diagonal matrix containing $\Omega_{v}$ and $\Omega_{w}$ (see [34]), and the trained emission distribution is represented as $\Phi^{\prime }=\left[\Phi_{v},\Phi_{w}\right]$. Thus, $\Gamma^{\prime }=\left\{\pi^{\prime },\Omega^{\prime },\Phi^{\prime }\right\}$ as shown in Algorithm 1.
**Algorithm 1:**Combined HMM.
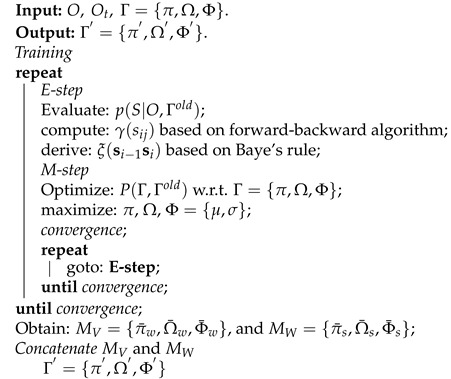


### 3.2. Prediction Algorithm

The prediction algorithm is obtained based on the trained HMM probabilities $\Gamma^{\prime }$ and the observation sequence $O_{t}$ of the RSS testing dataset. $O_{t}$ is first converted to Gaussian variables $O_{G}$ by computing the weight of each RSS data point in relation to the number of states using $\Phi^{\prime }$. Thereafter, the V-Alg [35] updates $O_{G}$ using parameters $\pi^{\prime }$ and $\Omega^{\prime }$ at time *t*. Hence, the most probable next AP can be identified from the corresponding hard decision (best path) of the updated $O_{G}$. To allow an unbiased handover decision from the VLC AP to WiFi AP, and vice versa, equal switching probability is given in the transition matrix for the V-Alg. Consequently, the best path value (Θ) derived from observed MT mobility at time *t* is compared to the average number of states N/2 such that the vertical handover decision is classified as follows:

(21) $$\Theta_{VHO}=\left\{\begin{array}{cc} VLC=0, & \Theta (t)>N/2\\ WiFi=1, & \Theta (t)\leq N/2. \end{array}\right.$$

This classification helps to prevent the ping-pong effect, which may occur due to the MT mobility and/or if the VLC LoS is blocked for a short period of time. The process of prediction is summarized in Algorithm 2.
**Algorithm 2:**Prediction Algorithm
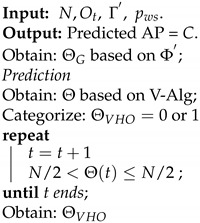


### 3.3. Model Performance Evaluation

The performance of the proposed HMM-based predictive VHO algorithm is analyzed using the RSS measurements and parameters described in Section 2. The VLC–WiFi dataset was visually inspected using MATLAB signal analyzer software. A 70% portion of the dataset is used for training, while the remaining 30% is set aside for validation and testing. The training samples were classified as VLC RSS and WiFi RSS, resulting in the formation of dual HMMs for both APs. The HMMs are ergodic forms, with each having two mixtures and three states. Therefore, the sequence of best paths Θ, each ending in 6 state at time *t*, is obtained for the dual model $\Gamma^{{}^{\prime }}$. Paths [4–6] and [1–3] were created during the user’s mobility and/or LoS blockage, corresponding to the different RSS levels of $M_{V}$ and $M_{W}$, accordingly. This implies that the paths {4; 1}, {5; 2}, and {6; 3} reflect the low, medium, and high RSS values, respectively.

The performance of the proposed HMM-based VHO technique is compared with the exiting techniques in the literature based on the following evaluation metrics:

1. Accuracy [36]:
  (22) $$A=\frac{TruePositive+TrueNegative}{TotalSample},$$
  where true positive is the number of times the output of HMM corresponds with the actual identified RSS values, while the true negative is the number of times the HMM predicts the wrong RSS values exactly as the actual wrong RSS value.
2. Mixed absolute percentage error [37]:
  (23) $$E=\frac{1}{M}\sum_{i=1}^{M}\frac{|\zeta_{i}-P_{i}|}{\zeta_{i}},$$
  where *M* is the number of predicted points, ζ is the actual RSS value, and P is the predicted RSS values. These metrics are used to obtain the difference between the actual measured value and the predicted value of the model.

## 4. Results and Discussion

In this section, the performance of the proposed HMM-based VHO prediction scheme is first evaluated using different time windows *w*. Thereafter, the handover performance of the proposed HMM algorithm is compared to the threshold-based technique [38] that uses a fixed limit to initiate handover, and state-of-the-art intelligent methods: the fuzzy controller-based approach in the related references [18,19,20,21] and the neural network frameworks in [17]. The same heterogeneous network environment on MATLAB is setup to perform a fair comparison of the methods.

### 4.1. Performance Analysis for Different *w*

The A and E performances of the model are evaluated by varying the values of *w* as one, three, five, and eight time slots. Table 3 shows the performance of the proposed HMM for different values of *w*. For a very small time window w=1, the observation frame is approximately 1 m, depending on the MT’s speed, which is not sufficient for the HMM to make accurate predictions, thus resulting to a low A performance of 78.60% and a high E value of 1.90%. Moreover, w=3 offers an A performance gain of 18.81%, 7.13%, and 10.92 compared to the 1, 5, and 8 time slots (1 time slot is equivalent to 1 s), respectively. In addition, w=3 yields the least E of 0.13% in comparison to 1.90%, 0.38%, and 0.79% for w=1, 5, and 8 time slots, respectively. The variations in the prediction performance of w=5 and 8 occur since the blockage duration in a VLC–LoS is usually for a short duration. In addition, w=5 and 8 correspond to long observation frames that may involve several user mobility changes in an indoor scenario. Hence, we set *w* to a value of three time slots for the HMM since it offers the best A and E performance and restricts user’s mobility to between 3 and 5 m.

**Figure 4:**
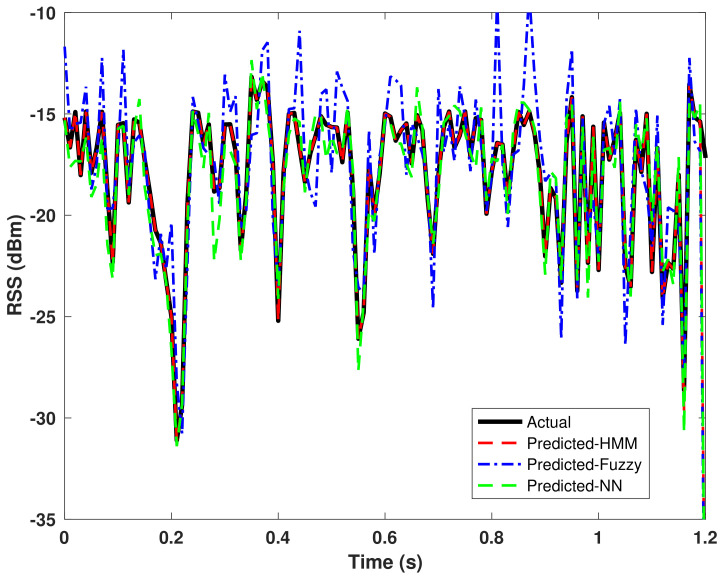
Actual versus Predicted RSS Values of the VLC–WiFi APs; HMM, Fuzzy, NN.

Additionally, the performance of the proposed HMM using w=3 is compared to that of the fuzzy-controller-based approach and NN techniques. The actual and predicted RSS values for the HMM, NN, and fuzzy-controller-based approaches are shown in Figure 4. Note that a high accuracy value is always desired to produce a limited error rate. The predicted RSS values of the VLC–WiFi APs using the proposed HMM match the actual RSS values with A=97.41% and E=0.13% compared to the NN-based algorithm having A=93.0% and E=0.18% and the fuzzy-controller-based approach yielding A=88.60% and E=0.92%. This indicates that the proposed HMM is more effective at predicting future RSS values in the indoor environment as it outperforms the NN and fuzzy logic approaches by accuracy performance gains of 8.81% and 4.41%, respectively. Furthermore, the proposed HMM algorithm has the lowest percentage error rate compared to the NN and fuzzy logic methods.

Likewise, Table 4 depicts the performance and computational time of the proposed HMM compared to the fuzzy-controller-based and NN approaches with w=3.

The results show that the fuzzy controller has the least computing time while yielding the worst prediction (accuracy and error rate) performance when compared to the HMM and NN. On the other hand, the computing time for training and testing in the NN model is higher compared to the HMM due to the number of hidden layer neural nodes. More so, the prediction errors in the NN method increases when very small network nodes are used. Therefore, six hidden layers were chosen as compared to the HMM, which utilizes a window size of three and less dimensional features. In this study, the number of neurons is set to six to minimize the NN’s final mean square error function result. The results in Figure 4 and Table 4 indicate that the proposed HMM technique provides a reasonable tradeoff between the computational complexity and prediction performance, thus making it a desired method for enhanced VHO prediction in the indoor scenario.

### 4.2. Handover Performance Evaluation

**Figure 5:**
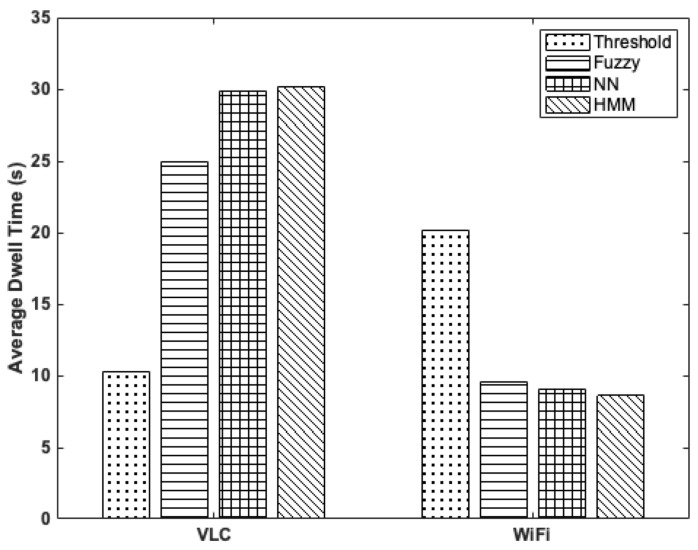
Average dwell time comparison of the VLC and WiFi systems using HMM, Fuzzy, NN, Threshold methods.

The average duration of time that the MT spends in a cell is evaluated and shown in Figure 5.

In the VLC AP (primary cell), the proposed HMM-based VHO prediction algorithm exhibits an average dwell time of 30.14 s compared to the NN model, fuzzy-based algorithm, and the threshold-based scheme with average dwell times of 29.86 s, 25 s, and 10 s, respectively. This implies that the proposed VHO algorithm enables the MT to stay longer on the VLC AP and thus reduces the ping-pong effect associated with the heterogeneous VLC–WiFi system. This is because the user’s movement has been restricted to 3 m; therefore, the proposed model can effectively optimize the probabilities of switching between the states in a given time. Additionally, the result shows that the proposed HMM-based VHO algorithm enables the MT to dwell on the WiFi AP (secondary cell) for an average time of 8.67 s compared to the NN algorithm with an average dwell time of 9 s, the fuzzy-based technique with an average dwell time of 9.58 s, and the threshold scheme with an average dwell time of 20.15 s. The threshold-based technique permits MT to switch between APs by choosing the one with a higher RSS value than the current one and the configured limit. In this study, RSS values of −35 dBm and −98 dBm are set as the thresholds for the VLC and WiFi APs, respectively. These limits are set to values above the minimum required RSS to allow more dwell time on the current link in service. Note that the proposed HMM and NN methods have about the same dwell time on the primary and secondary channels, however, with a higher computing complexity, as shown in Table 4.

**Figure 6:**
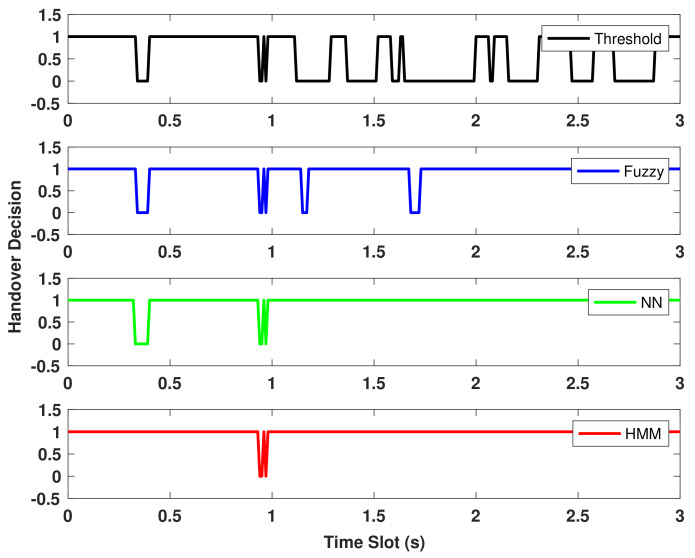
Handover decision comparison for proposed HMM, Fuzzy, NN, Threshold methods.

Furthermore, the average number of handovers in the heterogeneous VLC–WiFi system is investigated. Figure 6 shows the average number of handovers performed by the proposed HMM-based prediction algorithm, NN method, fuzzy-controller-based approach, and threshold-based technique.

The result shows that the threshold-based technique exhibits greater ping-pong handover in 3 s compared to the other schemes. This is because MT switches between APs whenever the RSS value is less than the average RSS value in the dataset. Otherwise, the MT continues to transmit using the same AP. More importantly, the MT appears to make a lot of switches in a short period of time because it has no knowledge of future RSS values or the user’s location. Moreover, the intelligent VHO prediction algorithms reduce the number of ping-pong handovers in the system based on dynamic decision-making schemes, thus reducing unnecessary switching. The proposed HMM method uses a forward–backward algorithm to calculate the probability of a sequence of RSS observations and then predicts the most-likely MT user position from the observed RSS sequence based on the V-Alg. Hence, the HMM reduces the amount of undesirable handover occurrences while being computationally efficient. The result shows that the HMM exhibits an average number of 3 handover events within 3 s, compared to the NN based approach having an average number of 4 handover events, the fuzzy-controller-based algorithm with around 6 handover events, and the threshold scheme with approximately 12 handover events.

## 5. Conclusions

In this article, an efficient vertical handover prediction scheme in a hybrid VLC–WiFi system is developed based on the HMM. The proposed scheme helps to resolve the issue of vertical handover caused by blockage and shadowing and user mobility in an indoor environment. It is also advantageous in situations where users may encounter frequent link failures due to ping-pong effects, which are usually associated with heterogeneous VLC–RF (WiFi) systems. To achieve this, two Gaussian HMMs are formed to model the VLC and WiFi using the unique RSS characteristics of the channels. The decision scheme utilizes the trained HMM probabilities and Viterbi decoding to select the best RSS value as the final switch and provide good user experience. The computational cost of HMM depends on the RSS feature dimension while the prediction performance is dependent on the time window at a given time. Therefore, PCA is used to extract effective RSS values from the huge datasets, which is adopted with HMM, thus reducing the computational complexity of the model. In addition, different window sizes are tested to select the one that maximizes the MT’s movement in a considerable observation frame. The results show that the proposed algorithm exhibits a high prediction accuracy and low mixed absolute percentage error performance by using a time window value of 3, which limits the user’s movement to between 3 and 5 m. Moreover, the results show that the proposed scheme improves the dwell time on a network and reduces the number of handover events as compared to the threshold-based handover scheme, the commonly used fuzzy-controller scheme, and the neural network technique. The neural network produces about the same results as the proposed HMM but with more computing complexity due to the number of hidden layer neural nodes. The proposed method can be applied to different network configuration while using more network characteristics.

## Figures and Tables

**Table 1:** VLC System Parameters.

Transmitter	
Room size (length × width × height)	3 m × 3 m × 1.5 m
$\vartheta_{1/2}$	$60^{\circ }$
LED Driving Current	300 mA
LED Modulation	Pulse Width Modulation: 50% duty cycle
Switching Frequency	100 Hz
Modulation Bandwidth	1 MHz
**Receiver**	
Optical Filter Wavelength	660 nm
TIA Bandwidth	240 MHz
Flourescent Frequency	50 Hz
Height of PD from floor	0.63 m
*A*	$10^{-4}$ m${}^{2}$
$\vartheta_{c}$	$60^{\circ }$
FFT size	256 samples
PD Responsivity	0.55 A/W
Sampling Frequency	128 kHz
Cutoff Frequency	100 MHz
$c(\theta_{r,t})$	10
$g(\theta_{r,t})$	1

**Table 2:** RSS Dataset Intervals.

Intervals	VLC	WiFi
High (dBm)	[−5~−13]	[−38~−63]
Medium (dBm)	[−14~−19]	[−64~−79]
Low (dBm)	[−20~−30]	[−80~−95]

**Table 3:** Performance analysis for different *w*.

*w*	A(%)	E(%)
1	78.60	1.90
3	97.41	0.13
5	90.28	0.38
8	86.49	0.79

**Table 4:** Performance analysis of the proposed HMM, fuzzy controller and NN; w=3.

Methods	A(%)	E(%)	Computational Time (s)
HMM	97.41	0.13	3.1667
NN	93.0	0.18	4.9875
Fuzzy	88.6	0.92	1.1394

## Data Availability

Datasets used and/or analyzed in this study are available from the corresponding author upon reasonable request.

## Abbreviations

The following abbreviations are used in this manuscript:

VLC	Visible light communication
LoS	line-of-sight
NLoS	non-line-of-sight
RF	radio frequency
QoE	quality of experience
AP	access point
RSS	received signal strength
HMM	hidden Markov model
WiFi	Wireless Fidelity
LED	light-emitting diode
MT	mobile terminal
HHP	homogeneous handover prediction
eNodeB	evolved NodeB
LTE	long-term evolution
VHO	vertical handover
PVHO	predictive vertical handover
IVHO	immediate vertical handover
DVHO	dwell vertical handover
CAD-VHO	channel adaptive dwell vertical handover
MDP	Markov decision process
AHP	analytical hierarchy process
MADM	multi-attribute decision making
CG	cooperative game
NN	neural network
ANN	artificial neural network
LVQNNs	learning vector quantization neural networks
WiMax	Worldwide Interoperability for Microwave Access
DC	direct current
PD	photo-detector
CSMA/CA	carrier sense multiple access with collision avoidance
PGM	probabilistic graphical model
ML	maximum likelihood
EM	expectation maximization
V-Alg	Viterbi algorithm
PIN	positive intrinsic negative
TIA	trans-impedance amplifier
AWGN	additive white Gaussian noise
PCA	principal component analysis
FoV	field of view
FFT	Fast Fourier Transform
PCs	principal components
SVD	Singular vector decomposition
LDA	linear discriminant analysis
FDA	Fisher’s discriminant analysis
DMD	dynamic mode decomposition
ISI	Intersymbol Interference
